# The Complete Genome Sequence and Comparative Genome Analysis of the High Pathogenicity Yersinia enterocolitica Strain 8081

**DOI:** 10.1371/journal.pgen.0020206

**Published:** 2006-12-15

**Authors:** Nicholas R Thomson, Sarah Howard, Brendan W Wren, Matthew T. G Holden, Lisa Crossman, Gregory L Challis, Carol Churcher, Karen Mungall, Karen Brooks, Tracey Chillingworth, Theresa Feltwell, Zahra Abdellah, Heidi Hauser, Kay Jagels, Mark Maddison, Sharon Moule, Mandy Sanders, Sally Whitehead, Michael A Quail, Gordon Dougan, Julian Parkhill, Michael B Prentice

**Affiliations:** 1 The Pathogen Sequencing Unit, The Wellcome Trust Sanger Institute, Wellcome Trust Genome Campus, Hinxton, Cambridge, United Kingdom; 2 Department of Infectious and Tropical Diseases, London School of Hygiene and Tropical Medicine, London, United Kingdom; 3 Department of Chemistry, University of Warwick, Coventry, United Kingdom; 4 Department of Microbiology, University College Cork, Cork, Ireland; 5 Department of Pathology, University College Cork, Cork, Ireland; Department of Energy Joint Genome Institute, United States of America

## Abstract

The human enteropathogen, *Yersinia enterocolitica,* is a significant link in the range of *Yersinia* pathologies extending from mild gastroenteritis to bubonic plague. Comparison at the genomic level is a key step in our understanding of the genetic basis for this pathogenicity spectrum. Here we report the genome of Y. enterocolitica strain 8081 (serotype 0:8; biotype 1B) and extensive microarray data relating to the genetic diversity of the Y. enterocolitica species. Our analysis reveals that the genome of Y. enterocolitica strain 8081 is a patchwork of horizontally acquired genetic loci, including a plasticity zone of 199 kb containing an extraordinarily high density of virulence genes. Microarray analysis has provided insights into species-specific Y. enterocolitica gene functions and the intraspecies differences between the high, low, and nonpathogenic Y. enterocolitica biotypes. Through comparative genome sequence analysis we provide new information on the evolution of the *Yersinia.* We identify numerous loci that represent ancestral clusters of genes potentially important in enteric survival and pathogenesis, which have been lost or are in the process of being lost, in the other sequenced *Yersinia* lineages. Our analysis also highlights large metabolic operons in Y. enterocolitica that are absent in the related enteropathogen, *Yersinia pseudotuberculosis,* indicating major differences in niche and nutrients used within the mammalian gut. These include clusters directing, the production of hydrogenases, tetrathionate respiration, cobalamin synthesis, and propanediol utilisation. Along with ancestral gene clusters, the genome of Y. enterocolitica has revealed species-specific and enteropathogen-specific loci*.* This has provided important insights into the pathology of this bacterium and, more broadly, into the evolution of the genus. Moreover, wider investigations looking at the patterns of gene loss and gain in the *Yersinia* have highlighted common themes in the genome evolution of other human enteropathogens.

## Synopsis

The goal of this study was to catalogue all the genes encoded within the Y. enterocolitica genome to help us better understand how this bacterium and related bacteria cause different diseases. There are currently genome sequences (complete gene catalogues) available for two other members of this bacterial lineage, which cause dramatically different diseases: Y. pseudotuberculosis, like *Y. enterocolitica,* is a gut pathogen (enteropathogen) causing gastroenteritis in humans and animals. Yersinia pestis mostly resides within blood (circulating or in fleas following blood meals) and lymph tissue. It causes bubonic plague in humans and animals, and is historically known as “The Black Death.” A three-way comparison of these genomes revealed a patchwork of genes we have defined as being species- or disease-specific and genes that are common to all three *Yersinia* species. This has provided us with important information on shared gene functions that define the two enteropathogenic yersinias and those that differentiate them. This will help us to connect what we know about the Y. enterocolitica lifestyle within the gut to the disease it causes and its genetic makeup. We have also provided further evidence of gene-loss by Y. pestis as it has evolved from Y. pseudotuberculosis into a more acute systemic pathogen. Similar patterns of gene loss are seen in other important pathogens such as Salmonella enterica serovar Typhi.

## Introduction

Y. enterocolitica is a globally distributed gastrointestinal pathogen that represents a key link in our understanding of how the three human pathogenic *Yersinia* species, *Y. enterocolitica, Y. pseudotuberculosis,* and *Y. pestis,* have evolved to produce diverse clinical manifestations. Like *Y. enterocolitica, Y. pseudotuberculosis* is an enteropathogen that is widely found in the environment, but it causes more severe clinical manifestations than Y. enterocolitica [1]. Y. pestis is primarily a rodent pathogen that is transmitted by the bite of an infected flea, and causes the often fatal systemic infection, bubonic plague [2]. Multilocus sequence analysis and DNA–DNA hybridization studies suggest that Y. enterocolitica and Y. pseudotuberculosis diverged within the last 200 million years and that Y. pestis is a clone of Y. pseudotuberculosis that has emerged within the last 1,500–20,000 years [3–5].

Since the pathogenic yersiniae diverged, Y. enterocolitica has evolved into an apparently heterogeneous collection of organisms encompassing six biotypes differentiated by biochemical tests (1A, 1B, 2, 3, 4, and 5) [6]. These in vitro biotypes group into three distinct grades of pathogen: a mostly nonpathogenic group (biogroup 1A); weakly pathogenic groups that are unable to kill mice (biogroups 2–5); and a highly pathogenic, mouse-lethal group (biogroup 1B) [6–9]. These biogroups have geographically distinct distributions, with biotype 1B being most frequently isolated in North America (termed the “New-World” strains), whereas biogroups 2–5 predominate in Europe and Japan (termed the “Old-World” strains) [10,11].

It is clear that DNA acquisition by lateral gene transfer has been fundamental in the emergence of the pathogenic yersiniae, all of which possess a 70-kilobase (kb) virulence plasmid (pYV) [12,13] and carry additional genetic factors located on the chromosome that are important for virulence [14–17]. However, current knowledge of the genetic repertoire that differentiates these strains is incomplete. Representatives of the two other human pathogenic *Yersinia* species, Y. pseudotuberculosis strain IP32953 (referred to as Y. pseudotuberculosis), and Y. pestis (strains CO92 [biovar Orientalis], KIM10+ [biovar Mediaevalis], and 91001 [biovar Microtis]; unless stated otherwise, all further references to Y. pestis relate to strain CO92), have been sequenced [18–21]. To define key steps in the evolution of the pathogenic yersiniae, we sought to define the genetic factors that were conserved in all of the pathogenic species from those that distinguish *Y. enterocolitica.* In addition, since Y. enterocolitica is a heterogeneous species we undertook microarray analysis aimed at relating the insights gained from the sequence data of strain 8081 biotype 1B to the other Y. enterocolitica biotypes.

## Results/Discussion

### General Features

**Figure 1:**
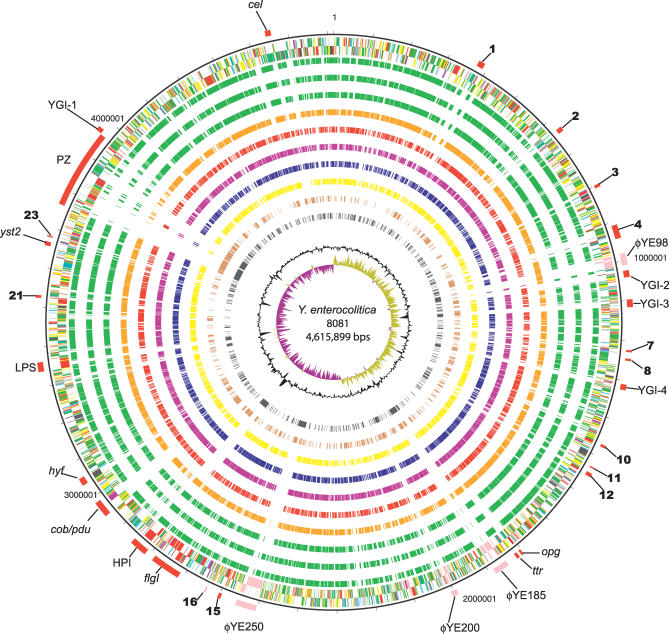
Circular Representation of the Y. enterocolitica Strain 8081 Chromosome The outer scale shows the size in bps. From the outside in, circles 1 and 2 show the position of CDSs transcribed in a clockwise and anticlockwise direction, respectively (for colour codes see below). Circles 3–5 (all CDSs coloured green) mark the position of Y. enterocolitica strain 8081 genes that have orthologues (by reciprocal FASTA analysis) in Y. pestis strains CO92, 91001, and KIM10+ and in (circle 6) Y. pseudotuberculosis strain IP32953 (CDSs coloured orange), respectively. Circles 7–10 show the Y. enterocolitica strain 8081 CDSs present (as detected by microarray) in all of the Y. enterocolitica isolates tested from biotype 1A (eight strains, red), biotype 2 (two strains, pink), biotype 3 (eight strains, blue), and biotype 4 (eight strains, yellow). Circle 11 shows CDSs unique to Y. enterocolitica strain 8081 (brown) compared with Y. pestis strain CO92 and Y. pseudotuberculosis strain IP32953 as determined by reciprocal FASTA analysis. Circle 12 shows CDSs unique to Y. enterocolitica strain 8081 (black) biotype 1B compared to all isolates of Y. enterocolitica biotypes 1A, 2, 3, and 4 as determined by microarray analysis. Circle 13 shows a plot of G + C content (in a 10-kb window) and circle 14 shows a plot of GC skew ([G − C]/[G + C] in a 10-kb window). Genes in circles 1 and 2 are colour-coded according to the function of their gene products: dark green, membrane or surface structures; yellow, central or intermediary metabolism; cyan, degradation of macromolecules; red, information transfer/cell division; cerise, degradation of small molecules; pale blue, regulators; salmon pink, pathogenicity or adaptation; black, energy metabolism; orange, conserved hypothetical; pale green, unknown; and brown, pseudogenes. The position of prophage elements (pink) and other important regions of difference (mentioned in the text) are marked (red). See Table 2 for a description. LPS, lipopolysaccharide biosynthetic genes.

The genome of Y. enterocolitica is very similar in size, number of predicted genes, and nucleotide composition to those of Y. pestis and Y. pseudotuberculosis (for a summary see Figure 1 and Table 1)*.* The most notable differences lie in the numbers of insertion-sequence elements and pseudogenes. Although the total number of insertion-sequence elements carried by Y. enterocolitica is lower than the other yersiniae, their diversity is greater, due to a recent expansion of a few elements in *Y. pestis* (see Table S1).

**Table 1:**
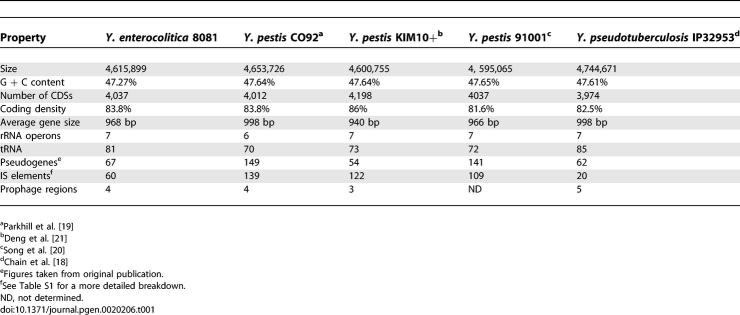
Properties of All the Published *Yersinia* Genomes

**Table 2:**
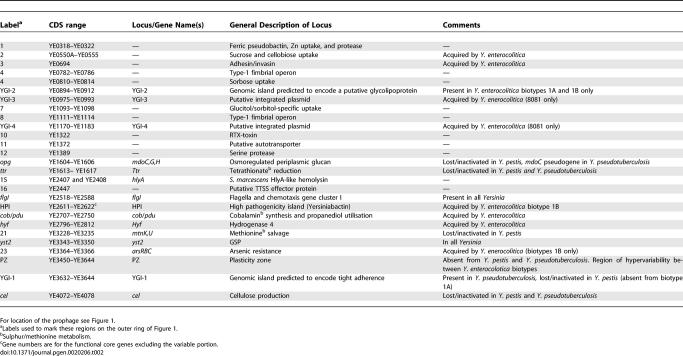
Significant Regions of Difference Identified in Chromosomal Gene Repertoire between the *Yersinia* Identified by Genome Sequencing and Microarray Analysis

Y. enterocolitica possesses a similar number of pseudogenes (67 coding sequences [CDSs]) to Y. pseudotuberculosis (62 CDSs). This is in contrast to *Y. pestis,* which is thought to have >140 chromosomal pseudogenes derived from point mutations, insertion sequence element insertions, large-scale rearrangements, and deletions, reflecting a marked change in lifestyle (associated with specific plasmid-acquisition events) [18,19]. This implies that Y. enterocolitica and Y. pseudotuberculosis have been stably maintained in a consistent niche [22].

**Figure 2:**
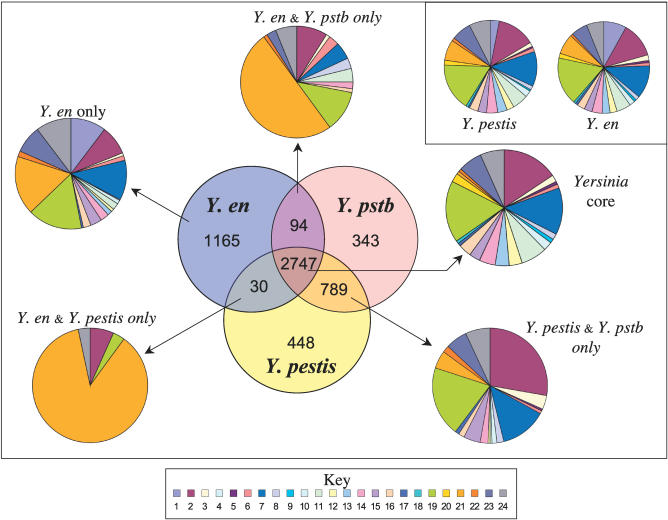
Distribution of Orthologous CDSs in Y. enterocolitica 8081, Y. pestis CO92, and Y. pseudotuberculosis IP32953 The Venn diagram shows the number of genes unique or shared between two other *Yersinia* species (see Materials and Methods). The associated pie charts show the breakdown of the functional groups assigned for CDSs in relevant sections of the Venn diagram. Colour code for the pie charts is as follows: hypothetical proteins (1); conserved hypothetical proteins (2); chemotaxis and motility (3); chromosomal replication (4); chaperones (5); protective responses (6); transport and binding proteins (7); adaptations to atypical conditions (8); cell division (9); macromolecule degradation (10); synthesis and modification of macromolecules (11); amino acid biosynthesis (12); biosynthesis of cofactors, prosthetic groups, and carriers (13); central intermediary metabolism (14); small-molecule degradation (15); energy metabolism (16); fatty acid biosynthesis (17); nucleosides and nucleotide biosynthesis and metabolism (18); periplasmic/exported/lipoproteins (19); ribosomal proteins (20); laterally acquired (including prophage CDSs) (21); pathogenicity and virulence (22); general regulation (23); and miscellaneous function (24). *Y. en, Y. enterocolitica* strain 8081; *Y. pstb, Y. pseudotuberculosis* strain IP32953; *Y. pestis, Y. pestis* strain CO92*.*

Although general features of the Y. enterocolitica genome are similar to those of the other sequenced *Yersinia,* there is considerable variation in gene repertoire. Reciprocal FastA searches were used to identify orthologous gene sets shared between Y. enterocolitica strain 8081*, Y. pestis* strain CO92, and Y. pseudotuberculosis strain IP32953 (Figure 2)*.* The yersiniae were found to share 2,747 core CDSs, with a significant number of CDSs being unique to Y. enterocolitica strain 8081 (∼29%)*, Y. pseudotuberculosis* strain IP32953 (∼9%), or Y. pestis strain CO92 (∼11%).

The number of CDSs shared exclusively between Y. enterocolitica and either Y. pseudotuberculosis or Y. pestis was initially surprising (see Figure 2). However, prophage accounted for a significant proportion of these CDSs. These phage-related CDSs are located in distinct gene clusters within different prophage-like elements and so these are unlikely to be true orthologues.

In addition to prophage-related CDSs, CDSs shared between Y. pseudotuberculosis and Y. enterocolitica and absent from *Y. pestis* fell into a range of other functional categories such as protective responses, adaptation to atypical conditions, and exported proteins (Figure 2). In contrast, CDSs found only in Y. enterocolitica and *Y. pestis* were either prophage-related or accounted for by differences in annotation. It is highly unlikely that both Y. pseudotuberculosis and Y. enterocolitica independently acquired these functions since the divergence of Y. pseudotuberculosis and Y. pestis; these functions have therefore probably been lost by Y. pestis since diverging from Y. pseudotuberculosis. To investigate this further, we scrutinised the genomic context of the CDSs and identified the corresponding regions in *Y. pestis.* For some of the *Y. pseudotuberculosis-* and Y. enterocolitica-specific functions, all indications of their presence in Y. pestis have been lost. However, in several instances it was possible to identify remnants of these regions in Y. pestis. These CDSs may represent ancestral functions important for an enteric lifestyle, but which subsequently became redundant for Y. pestis. Alternatively, given the high virulence potential of Y. pestis, some of these gene changes (gene losses) may be examples of pathoadaptive mutations [23].

We performed the same analysis for the Y. enterocolitica-specific loci and were able to identify deletion scars (gene remnants) for some of these regions that were apparent in both Y. pestis and Y. pseudotuberculosis; all of these loci are detailed below (summarised in Table 2).

### Evidence of Ancestral *Yersinia* Gene Functions in the Y. enterocolitica Genome

#### Metabolism and adaptation.

Within the CDSs shared exclusively by Y. enterocolitica and *Y. pseudotuberculosis,* there are two entire metabolic pathways that have apparently been completely lost by *Y. pestis:* the methionine-salvage pathway and the osmoregulated periplasmic glucan (OPG) biosynthetic pathway.

The methionine-salvage pathway recycles the sulphur-containing compound, methylthioadenosine (MTA), formed during spermidine and spermine synthesis, and as a byproduct of *N*-acylhomoserine lactone production. MTA is recycled back to methionine, which can be further metabolised to produce S-adenosylmethionine, an essential reactant in several methylation reactions (see [24] and references therein).

The methionine-salvage pathways are conserved and appear to be intact in both Y. enterocolitica and Y. pseudotuberculosis. In *Y. enterocolitica,* the CDSs involved encode MtnK (kinase, YE3228), MntA (isomerase, YE3230), MtnD (dioxygenase, YE3231), MtnC (bifunctional enolase/phosphatase, YE3232), MtnB (dehydratase, YE3233), MtnE (transaminase, YE3234), and MtnU (possible regulator, YE3235). In addition, there is a second unlinked locus encoding a nuclease (MtnN, YE0739). Therefore, the Y. enterocolitica methionine salvage pathway is similar to that of *Klebsiella pneumoniae,* with a two-stage conversion of MTA into methylthioribose-1-phosphate and a bifunctional MtnC [25]. In *Y. pestis,* all of the CDSs encoded in the *mtnK–mtnU* locus are missing (presumably deleted)*.* However, the *mtnN* gene has been retained in Y. pestis (YPO3384 in strain CO92, YP0301 in strain 91001, and y0802 in strain KIM10+) and remains intact and in the same genetic context as the *Y. enterocolitica mtnN* gene. It is known that in nutrient-rich environments and in the presence of low concentrations of dioxygen, facultatively anaerobic bacteria, such as *Escherichia coli,* simply convert MTA into methylthioribose, using MtnN, and excrete it from the cell. This is likely to be the case for *Y. pestis,* too, since growth outside of the nutrient-rich environment of the host is unnecessary for its current lifestyle.

OPG is an important constituent of the outer membrane in many Proteobacteria. It was originally identified as being involved in osmoprotection [26]. However, the function of OPG is more complex, since OPG mutants are highly pleiotropic, with defects in virulence, biofilm formation, resistance to antibiotics, and a hypersensitivity to bile salts. The *Y. enterocolitica opg* cluster is composed of *mdoC*, *mdoG,* and *mdoH* (YE1604–YE1606, respectively). Orthologues of all the *opg* genes are present in Y. pseudotuberculosis (YPTB2493–YPTB2495) [27], but *mdoC* carries multiple nonsense mutations. This entire *opg* cluster is absent from Y. pestis and is thought to have been deleted, although no detectable remnants remain*.*

The loss of the OPG cluster by *Y. pestis,* and its retention by the two enteropathogenic *Yersinia,* suggests that it remains important for their enteric lifestyle. However, although Y. pseudotuberculosis maintains these CDSs, the loss of a functional *mdoC* gene suggests that the Y. pseudotuberculosis OPG is nonsuccinylated, and so its function may differ from that of Y. enterocolitica.

Two other complete Y. enterocolitica metabolic pathways have apparently been lost from Y. pseudotuberculosis and *Y. pestis,* leaving deletion scars behind. These include the cellulose *(cel)* biosynthetic operon (YE4072–YE4078), which is highly similar in gene content and sequence to that carried by most *Salmonella*. The only remaining *cel* CDS in Y. pseudotuberculosis and *Y. pestis* is *bcsZ,* encoding endo-1,4-β-glucanase. Although *bcsZ* appears intact in Y. pseudotuberculosis (YPTB3837), the *Y. pestis bcsZ* orthologue carries a frameshift mutation. An identical mutation is present in the *bcsZ* genes in all of the sequenced Y. pestis isolates.

Cellulose production by bacteria is also associated with protection from chemical, as well as mechanical, stress [28]. In *Salmonella,* the cellulose biosynthetic operon is thought to constitute a transferable module that was acquired by an enterobacterial ancestor as well as a range of other unrelated bacteria [28]. *Salmonella* produce cellulose in concert with thin aggregative fimbriae to form an inert and highly hydrophobic extracellular matrix. It has been suggested that the protection afforded by this matrix increases retention time of the bacterium in the gut and so offsets the high-energy cost incurred in its production [28]. Cellulose production is presumably redundant for Y. pestis in its new lifestyle. However, why this operon should have been lost by Y. pseudotuberculosis is not as clear. It may reflect niche differences within the enteric environment between the two enteropathogenic *Yersinia* species, such as the length of time these bacteria reside extracellularly exposed in the gut.

The other pathway deleted from the Y. pseudotuberculosis lineage is tetrathionate respiration. The ability to respire the sulphur-containing compound tetrathionate is used as an identifying trait for Y. enterocolitica [29] and is facilitated by the tetrathionate reductase–gene cluster (*ttr*, YE1613–YE1617). The *ttr* genes appear to have been completely lost from Y. pestis apart from a remnant (identical in all isolates) of *ttrR* (encoding a two-component regulator governing the tetrathionate operon). All of the *ttr* genes are missing from Y. pseudotuberculosis. The retention of the complete *ttr* cluster by Y. enterocolitica is interesting because, uniquely amongst the *Yersinia*, Y. enterocolitica possess the coenzyme B_12_–biosynthetic (*cbi*) and 1,2-propanediol-degradation (*pdu*) gene clusters located on a single 40-kb genomic island (YE2707–YE2750) [30,31]*.* This island is inserted between genes that are adjacent in Y. pestis and *Y. pseudotuberculosis.* Coenzyme B_12_ is known to be produced only under anaerobic conditions [30] and is essential for the degradation of 1,2-propanediol as a source of carbon and energy [30,31].

*Salmonella* possesses *cbi*, *pdu,* and *ttr* gene clusters that are highly related to those of *Y. enterocolitica.* Like Y. enterocolitica, *Salmonella* only produces endogenous B_12_ anaerobically and under those conditions the energetically efficient anaerobic degradation of 1,2-propanediol proceeds with tetrathionate acting as a terminal electron acceptor facilitated by the gene products of the *ttr* genes [31]. This is likely to also be true for Y. enterocolitica and may therefore explain why the *ttr* operon has been retained in this species. As has been proposed for *Salmonella* [31], this also suggests that 1,2-propanediol is an important source of energy for Y. enterocolitica (and not Y. pseudotuberculosis). The horizontal transfer of the *cob/pdu* operon has previously been noted as a feature in *Salmonella* and E. coli divergent evolution [32,33]*.*

#### Adhesion.

In addition to revealing the loss of complete biochemical pathways, the Y. enterocolitica sequence suggests more subtle examples of loss of function in Y. pestis. All the pathogenic yersiniae possess a cluster of 13 CDSs on a genomic island displaying a lower G + C content (35%; compared with genome average of 47%) that we have denoted as *Yersinia* Genomic Island 1 (YGI-1, YE3632–YE3644 [Figure 1]). YGI-1 is highly related in sequence and gene content to a family of genomic islands, denoted *tad* loci (tight adherence), present in diverse bacterial and archaeal species, including *Actinobacillus actinomycetemcomitans, Pyrococcus abyssi,* and Y. pestis [34,35]. The *tad* locus of *A. actinomycetemcomitans,* a human pathogen causing endocarditis and periodontitis, has been shown to be important for virulence by encoding the biosynthesis and transport of pili involved in tight, nonspecific adherence [34,36]. In *Y. pestis,* it has been speculated that the *tad* genes are important for the colonisation of the flea [36]. However, our data makes this hypothesis unlikely. A comparison of all the *Yersinia* YGI-1 islands shows that whilst these regions are intact in Y. enterocolitica and *Y. pseudotuberculosis,* the Y. pestis YGI-1 gene cluster has been truncated by the insertion of IS*1541* elements that have resulted in the deletion of the essential pilin gene, *flp*. Furthermore, all of the sequenced Y. pestis isolates carry an identical frameshift mutation in *rcpA* (OutD-like type II secretion protein; YPO0692 in strain CO92, YP3007 in strain 91001, and Y3485 in strain KIM10+), predicted to ablate function. This suggests that the loss of this phenotype occurred only once and soon after Y. pestis and Y. pseudotuberculosis diverged. Moreover, since it is predicted that the Tad pilus would be exposed on the surface of the cell, like the loss of YadA [37], this may be another example of a key mutational event that was selected for by the change in lifestyle of Y. pestis. Consequently, far from being an adaptation to life within the flea, this cluster is likely to be important for enteropathogenicity, explaining why YGI-1 remains intact in Y. enterocolitica and *Y. pseudotuberculosis.*

### Y. enterocolitica Unique CDSs: Functions Acquired Since the Divergence of the Species

Orthologue searches revealed that more than one quarter of the Y. enterocolitica CDSs are absent from the other sequenced *Yersinia* species. If these CDSs are viewed in the context of the genome, it is evident that many are found in clusters ranging from ∼2–200 kb (Figure 1) and fall into a range of functional categories (Figure 2). Collectively, these species-specific loci contribute to virulence (plasticity zone [PZ]) and significantly broaden the metabolic capability (the hydrogenase operons and cobalamin and propanediol gene clusters, discussed above) of *Y. enterocolitica,* and consequently may provide clues as to how Y. enterocolitica adapted to its current niche (discussed below).

#### Plasticity zone: A key locus for high pathogenicity.

**Figure 3:**
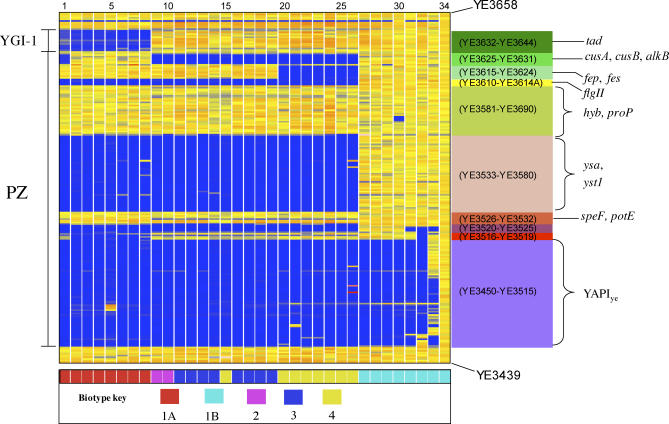
Microarray Analysis of the Plasticity Zone of 34 Isolates of Y. enterocolitica Biotypes 1A, 1B, 2, 3, and 4 Microarray analysis of the genomic DNA from 34 Y. enterocolitica isolates, representing five biotypes, constructed using GeneSpring version 6.1 software. Data is presented for the CDS in the range of YE3439–YE3658 including the PZ and YGI-1, as marked (left side). Each numbered column represents the results from a different Y. enterocolitica strain: 1, 09/03; 2, 12/02; 3, 208/02; 4, 35/03; 5, 77/03; 6, 30/02; 7, 81/02; 8, 14/02; 9, 119/02; 10, 212/02; 11, 218/02; 12, 231/02; 13, 56/03; 14, 16/03; 15, 209/02; 16, 149/02; 17, 177/02; 18, 153/02; 19, 202/02; 20, 7/03; 21, 135/02; 22, 8/03; 23, 190/02; 24, 220/02; 25, 227/02; 26, 201/02; 27, Y30; 28, Y73; 29, Y89; 30, Y71; 31, Y68; 32, Y70; 33, Y69; and 34, 8081 (control). See Table S2 for details. The colour-coded biotype key for each isolate is shown at the bottom. Each row represents an individual gene within this region. Coloured blocks (right side) have been used to highlight groups of CDSs showing differing distributions between isolates. The range of CDSs encoded within these blocks is shown (in brackets). Also marked are the relative positions of interesting CDSs or loci that have been mentioned within the body of this article. Blue CDSs correspond to those genes that are considered absent/divergent, and yellow CDSs correspond to genes that are assigned present/conserved. Grey indicates data not obtained.

The PZ is the largest region of species-specific genomic variation found within the Y. enterocolitica genome. It is bounded on one side by a tRNA-*phe* gene and accounts for ∼16% of the Y. enterocolitica unique CDSs (an ∼199 kb locus extending from 3,761,922–3,960,673 bps and encoding 186 CDSs [Figure 1]). The PZ is unlikely to have been acquired during a single event and is more likely to have arisen through a series of independent insertions at this site. Several discrete functional units are identifiable within this region, some of which are known to be mobile or sporadically distributed in other bacteria, and some of which are flanked by repeat sequences. These include a region highly similar to the Y. pseudotuberculosis adhesion pathogenicity island (YAPI_ytb_ [38], which we have denoted YAPI_ye_), type III (*ysa*) and type II (*yst1*) secretion system clusters, and several metal-uptake operons and resistance-gene loci (Figures 1 and 3).

#### Within the plasticity zone: YAPI_ye_.

The Y. enterocolitica YAPI_ye_ is located between 3,761,992–3,828,092 bps and is flanked by an intact and partial copy of the tRNA^phe^ gene, associated with the integration of this element into this site. In *Y. pseudotuberculosis,* YAPI_ytb_ encodes a type IV pilus operon shown to be important for virulence [38]. YAPI_ye_ (66 kb) is significantly smaller than YAPI_ytb_ (98 kb [38]), with a conserved core carrying the type IV pilus operon and encoding plasmid-related functions, as well as a variable region. The variable portion of YAPI_ytb_ is predicted to encode various metabolic functions and a type I restriction/modification system [38], whereas this region of YAPI_ye_ encodes a possible hemolysin (YE3454), a toxin/antitoxin system (YE3480 and YE3481), and an arsenic-resistance operon (YE3472–YE3475). Both the arsenic-resistance operon and the type IV pilus cluster are highly similar to those on the S. typhimurium plasmid R64. Arsenic resistance appears to be important for Y. enterocolitica strain 8081 since there is a second chromosomal arsenic-resistance operon (YE3364–YE3366) outside of the PZ, similar to the chromosomally encoded *E. coli arsRBC* operon [39], and a different transposon-borne arsenic-resistance operon carried on pYV has been reported from low virulence European strains of Y. enterocolitica [40]. Selection for arsenic resistance in Y. enterocolitica is believed to reflect intensive treatments of pigs with arsenical compounds in the pre-antibiotic era to protect them from diarrhoea caused by Serpulina hyodysenteriae [40].

The *Yersinia* YAPI islands share extensive similarity in sequence, gene content, and gene arrangement with the S. typhi pathogenicity island, SPI-7 [41–43], as well as a broader family of genomic islands found in a diverse set of bacteria [41,44,45].

#### Within the plasticity zone: Secretion systems.

In addition to the Yop type three secretion system (TTSS) encoded on pYV, the Y. enterocolitica PZ carries a second TTSS, denoted as Ysa [46,47]. The *Y. enterocolitica ysa* operon is composed of 32 CDSs (YE3533 [*acpY*]–YE3561[*ysrS*]) and is known to be important for pathogenicity, as *ysa* mutants show a reduced virulence phenotype [46].

The PZ also encodes (YE3564–YE3575), a general secretion pathway (GSP)–like system, denoted as Yst1 [17]. Like Ysa, mutants defective for the Yst1-secretion system were found to be impaired in colonisation when introduced by the oral route of infection [17]. In addition, Y. enterocolitica 8081 possesses a second GSP cluster, denoted as Yst2 (Figure 1) [17], which is located outside of the PZ region and is common to both Y. pestis and Y. pseudotuberculosis.

#### Within the plasticity zone: Niche adaptation.

The Y. enterocolitica PZ also carries several other gene clusters capable of conferring survival benefits in the gut or wider environment. These include the hydrogenase 2 biosynthetic operon (discussed below), an orthologue of the gene encoding the betaine/proline transporter, ProP (YE3594), a bifunctional protein with roles in both osmoprotection and osmoregulation, and a chitinase (YE3576) that could be secreted by Yst1 [17]. Other CDSs involved in metal uptake and resistance are present in this region. These include the ferric enterochelin operon *fepBDGC fes* and *fepA* (YE3618–YE3624 and [48]; note that *fepA* is a pseudogene, a system highly similar to the ferrichrome transport system, *fhu* (YE3583–YE3586), from Bacillus subtilis, YE3629 and YE3630, which are predicted to encode proteins similar to the E. coli silver and copper transporting efflux system, CusA and CusB [49]. Also noteworthy is YE3631, which encodes a product highly similar to the E. coli AlkB protein, which confers resistance to DNA-alkylating agents [50].

Gene loss is also evident in the PZ. Remnants of an ancestral enteric flagella cluster termed *flgII* [51] are present: YE3610 (lipoprotein), YE3610A (flagella protein, pseudogene), YE3611 (regulator [pseudogene]), and YE3614A (flagella regulator [pseudogene]) are orthologues of CDSs found bordering or within the Y. pestis flagella cluster II. The Y. enterocolitica flagella cluster I (2,711,620–2,787,043 bps) remains intact and is known to be functional and important for virulence [52]*.* However, *Y. pestis,* which, unlike *Y. enterocolitica,* is nonmotile, has retained both ancestral flagella clusters, albeit with some degeneracy [19].

#### Y. enterocolitica hydrogenase loci: Colonisation of the gut.

The ability to exploit locally generated hydrogen as a source of energy has been recently shown to be essential for colonisation of the gut, and for the virulence of enteric bacteria such as *Salmonella* and *Helicobacter* [53–55]. The Y. enterocolitica unique gene-set encodes two [NiFe]-containing hydrogenase complexes, Hyd-4 and Hyd-2, encoded within the *hyf* locus (YE2796–YE2812, encoding orthologues of E. coli genes *hypCA, hyfABCDEFGHIJK, hydN, fdfH, hyfR,* and *focB*) and the *hyb* locus (YE3600–YE3609, encoding orthologues of *E. coli hypFED, hybG, hypB, hybFEDCBAO*), respectively.

In *E. coli,* Hyd-2 acts in a respiratory capacity through the oxidation of molecular hydrogen [56]. Hyd-4 forms a complex with formate dehydrogenase H (Fdh-H), constituting formate hydrogen-lyase system 2 (Fhl-2). Three subunits of Hyd-4 (*hyfDEF*) are thought to facilitate the translocation of protons across the cytoplasmic membrane [57], thereby generating a proton gradient that can then be used to generate energy, mainly used to take up amino acids for more rapid growth (for a review see [58]).

The two Y. enterocolitica hydrogenase clusters are extremely compact, encoding all of the CDSs essential for the functioning and maturation of Hyd-4 and Hyd-2. This is not true of other enteric bacteria described to date, in which these functions are distributed over several different hydrogenase clusters and/or are dispersed throughout the genome. There is no evidence of the *hyf* and *hyb* loci in the Y. pestis and Y. pseudotuberculosis genomes. Coupled with their compact nature, this may suggest that they have been acquired by *Y. enterocolitica,* despite the absence of any obvious mobility genes in these clusters.

#### Prophage and other regions of difference.

As noted in the genomes of most other sequenced enteric bacteria, much of the Y. enterocolitica novel DNA is composed of prophage-like elements ([59,60]; Figure 1; locations 981,223–1,011,295, 1,849,792–1,887,236, 1,991,720–2,007,210, and 2,503,099–2,554,665, denoted as ΦYE98, ΦYE185, ΦYE200, and ΦYE250, respectively). All of the Y. enterocolitica prophage carry what appear to be “cargo genes,” which are not essential for phage replication but potentially functional in a lysogenic phase. Prophage cargo genes are involved in DNA methylation and regulation, as well as in restriction and modification; the restriction enzyme YenI (YE1808) [61] lies within a low G + C region of ΦYE200. Interestingly, considering the niche differences and diversity of prophage, Y. pestis carries a prophage that is highly related to ΦYE250 and Y. pseudotuberculosis carries prophage regions highly similar to ΦYE98 and ΦYE185 (Figure 1). These are not present in the same chromosomal context and are likely to be independent acquisitions.

In Y. pestis, the prophage resembling ΦYE250 (DNA identity 80%–90%) has been linked to the presence of noncoding chromosomal regions of clustered regularly interspaced short palindromic repeats (CRISPR loci, comprising direct repeats, from 21 to 37 bp, interspersed with similarly sized nonrepetitive sequences or spacers), also found in Y. pseudotuberculosis [62]. Most of the spacer sequences are thought to have been actively captured from this prophage by an unknown mechanism, and the CRISPR locus is thought to represent a defence system against bacteriophage [62]. Interestingly, four of the 31 described spacer sequences from the three Y. pestis CRISPR loci [62] are also present in the Y. enterocolitica prophage ΦYE250. Using a standard CRISPR detection method [63], we have not found a CRISPR locus in the nucleotide sequence of Y. enterocolitica 8081*.* Specific CRISPR-associated (Cas) proteins [64] corresponding to the Y. pestis genes YPO2462–8 are not present in *Y. enterocolitica.* Therefore, either the *Y. pestis–Y. enterocolitica* common ancestor possessed CRISPR loci lost in Y. enterocolitica 8081 evolution, or an active process has been occurring in Y. pseudotuberculosis and Y. pestis following acquisition of a CRISPR progenitor [65].

There are several other genomic loci that show a phylogenetically restricted distribution. These include a novel locus, composed of 13 CDSs (YE0894–YE0912), which we have denoted *Yersinia* genomic island 2 (YGI-2) (see Figure 1). YGI-2 is highly conserved as a genomic island in a wide range of *Enterobacteriaceae,* including the phytopathogen Erwinia carotovora subsp. *atroseptica* [44], enterohaemorrhagic E. coli 0157:H7 [66], and uropathogenic E. coli CFT073 [67], as well as in the probiotic E. coli strain Nissle [68]. Notably, this island is missing in E. coli K12 (unpublished data).

YGI-2 has a low G + C content (44.62 %) and is located alongside a tRNA^asp^ gene, characteristic of horizontal gene transfer, although there are no obvious mobility functions encoded on this island. The CDSs within this cluster appear to encode the biosynthesis, modification, and export of an outer membrane anchored glycolipoprotein, the function of which is unclear.

Additionally, there are several other notable genomic loci in this category that carry CDSs predicted to encode an RTX-toxin; an adhesin; sugar-, iron-, and zinc-uptake systems; fimbriae; and two loci that resemble integrated plasmids (see Table 2). Both of the putative integrated plasmids have an atypical G + C content; the first is inserted alongside the stable RNA *ssrA* gene (tmRNA, denoted as YGI-3, located 1097155–1116114 bps) and flanked by 14 bp direct repeats and the second element (denoted as YGI-4, located at 1308551–1323148 bps, see Figure 1 and Table 2) has inserted into YE1169, leaving an intact copy on one side and a partially duplicated copy (YE1184) on the other side of the element.

#### Microarray analysis of *Y. enterocolitica* biotype–specific variation.

Considering the range of different Y. enterocolitica biotypes and the differences they display in their pathogenicity, it was important to define those Y. enterocolitica strain 8081 genetic functions that are characteristic of the species as a whole and those that are strain- or biotype-specific. Microarray data for the genomic DNA of 34 Y. enterocolitica isolates, including 26 UK isolates of biotypes 1A, 2, 3, and 4 and eight US isolates of biotype 1B (including 8081), were used in this analysis and represented a subset of data taken from a much larger phylogenomic study [69] using a microarray based on Y. enterocolitica strain 8081 (This data is summarised in Figures 1 and 3).

The microarray data confirmed that several of the important metabolic regions detailed above were present in all biotypes tested and so are likely to represent key factors for niche adaptation by this enteropathogen. These include the two hydrogenase gene clusters *(hyb* and *hyf),* the cobalamin synthesis *(cob)* and propanediol utilisation operons *(pdu),* the gene cluster encoding cellulose biosynthesis *(cel),* tetrathionate respiration *(ttr),* and the OPG cluster *(opg).*

The most obvious biotype-specific regions shown by the Y. enterocolitica 8081 microarray were the four prophages. None of the prophage genes were conserved in the non- (biotype 1A) or mildly pathogenic (biotypes 2, 3, and 4) Y. enterocolitica (Figure 1). In contrast, the degenerate prophage, ΦY200, was fully represented in all 1B isolates except Y69 and Y70, where it was partially detected (unpublished data). Prophage sequences highly related to ΦYE98 were present in biotype 1B isolates Y69 and Y89, and Y71 harboured most genes from ΦYE185. Prophage ΦYE250 was unique to strain 8081 and is likely to be a recent acquisition, perhaps explaining the absence of a CRISPR locus, as discussed above.

The largest single Y. enterocolitica strain 8081–specific locus, seen through whole genome sequence comparisons with other yersiniae, was the PZ*.* In addition to showing species specificity, microarray analysis revealed that the PZ also showed a marked biotype-specific distribution, consistent with it being a region of hypervariability (Figure 3). Moreover, it was notable that the different subregions of the PZ showed clear biotype delineations, making it suitable for a PCR-based typing scheme.

Two regions within the PZ were common to all of the Y. enterocolitica isolates. The first region encoded the hydrogenase 2 cluster (*hyb*) and a second locus is predicted to encode SpeF and PotE, which are involved in polyamine uptake in other bacteria. YAPI_ye_, the TTSS *ysa,* and GSP *yst1* were all restricted to highly pathogenic 1B biotypes, consistent with previous findings [17,38,47]. Interestingly, we only detected the presence of YAPI_ye_ in one other Y. enterocolitica 1B isolate (Y69) in addition to 8081, and in this instance only CDSs predicted to encode the type IV pilus were present. The YAPI type IV pili genes lie within the core region of this family of mobile genetic elements [38], suggesting that a distinct YAPI_ye_ element with a different gene complement from that in 8081 may exist in this strain.

Also within the PZ, the ferric enterochelin operon was detected in all biotypes tested, except for strains of biotype 4 (consistent with previous results) [17,47,48], and CDSs YE3624–YE3630, which are predicted to encode several metal-resistance functions, were restricted to biotypes 1A and 1B (see Figure 3).

Notable biotype-specific regions outside of the PZ included genomic islands YGI-1 (*tad* genes), and YGI-2. YGI-2 was detected in biotypes 1A and 1B only (Figure 1), whilst YGI-1 was restricted to the pathogenic Y. enterocolitica biotypes (1B and 2–4; Figure 3). Since YGI-1 is present in all of the other Y. enterocolitica biotypes, and indeed the other pathogenic *Yersinia* species, it reinforces the view that this locus is important for enteropathogenicity and suggests that it has been lost from the biotype 1A lineage.

Using the microarray data, we determined that there were 992 CDSs present in Y. enterocolitica strain 8081 (biotype 1B) that were not detected in the biotypes 1A, 2, 3, and 4 isolates tested (Figure 1). Within this gene set, 406 CDSs were represented in all the other members of biotype 1B tested. Furthermore, 119 CDSs were unique to Y. enterocolitica strain 8081, as they were not detected in any of the other Y. enterocolitica isolates tested by microarray (Listed in Table S3).

Consistent with previous results, the biotype 1B–specific CDSs included the CDSs within the high-pathogenicity island and several of the regions located within the PZ, as discussed above. Other virulence-associated functions in this group include the Serratia marcescens HlyA-like hemolysin and activator (YE2407 and YE2408, also present in Y. pseudotuberculosis and Y. pestis) an autotransporter (YE1372), a serine protease (YE1389), and a putative TTSS effector protein (YE2447) that is highly similar (91% amino acid–sequence identity) to the Shigella flexneri TTSS effector protein OspG, which in S. flexneri is a protein kinase that has been shown to interfere with the innate immune response [70]. The arsenic-resistance operon (YE3364–YE3366) located outside of the PZ is also restricted to biotype 1B isolates. Interestingly, the integrated plasmid region (YGI-4) is variably present in several other 1B isolates (Y69 and Y30, unpublished data).

Of the CDSs that were found by microarray analysis to be unique to the sequenced strain 8081, the majority (104/119) were clustered, constituting ΦYE250, one of the two proposed integrated plasmid regions: YGI-3, the putative hemolysin (YE3454), and the variable portion of the YAPI_ye_, encoding the arsenic-resistance operon (Figure 3). It is likely that these elements represent the most recent acquisition events in this strain and this underlines the fact that lateral gene transfer continues to be an important source of new genetic material within the yersiniae.

## Conclusions

The genome of Y. enterocolitica and its comparison with the genomes of Y. pseudotuberculosis and Y. pestis reveal fascinating insights into gene loss and acquisition that have occurred since these yersiniae diverged. We identified *Y. enterocolitica–*specific genes, some of which showed evidence of previous loss from both Y. pestis and Y. pseudotuberculosis. We also identified loci that were putative enteropathogenic yersinia–specific genes retained by Y. enterocolitica and Y. pseudotuberculosis but lost by Y. pestis (Table 2)*.*

The core set of genes encoding orthologous proteins shared by Y. enterocolitica strain 8081*, Y. pestis* strain CO92, and Y. pseudotuberculosis strain IP32953, defined in this study by reciprocal FastA analysis (2,747 CDSs), is much higher than the number of core genes detected in all isolates of Y. enterocolitica by comparative genome hybridisation (894 CDSs) [69]. This can be explained either by a higher level of variation found within the Y. enterocolitica strains compared with that seen within Y. pestis and Y. pseudotuberculosis [71], or, more likely, that the number observed in [69] represents measurement of gene divergence rather than complete gene loss and so is an underestimation due to the constraints of comparative genome hybridisation analysis.

Microarray data was instrumental in identifying which of the metabolic functions identified from the sequenced strain could be considered core Y. enterocolitica functions. These data was then used to strengthen the comparison of the metabolic capabilities identified in the genome sequence of Y. enterocolitica strain 8081 with those of the other sequenced pathogenic Yersinia sp., identifying significant metabolic pathway differences.

Metabolic pathway defects long recognised in Y. pestis compared with Y. pseudotuberculosis [12] have suggested that there is a change in Y. pestis metabolism, triggered by the temperature difference between the flea and mammalian host. From the perspective of enteropathogenic yersiniae, we can identify another pathway that has been lost from *Y. pestis,* involving methionine salvage, correlating with its amino acid–rich blood environment. Methionine salvage–pathway enzymes can produce carbon monoxide [24], a molecule capable of affecting host gut signalling pathways [72], so there may be an additional nonnutritional advantage for this pathway in enteric pathogens. The presence of this pathway from the perspective of the enteropathogenic *Yersinia* is also interesting because it may present a target for antimicrobial chemotherapy [24].

 The loss of function in Y. pestis of many genes associated with enteric pathogenicity is widely accepted, but rather more surprising was the apparent loss by Y. pseudotuberculosis of several presumptive enteric adaptation functions maintained in *Y. enterocolitica.* These include the OPG and cellulose biosynthetic genes and the differences in polyamine uptake and metabolism. All these functions are associated with protection from physical and chemical stress, and their loss therefore suggests that Y. enterocolitica occupies a significantly different niche than *Y. pseudotuberculosis,* which is more exposed to the conditions experienced within the gut lumen, and is perhaps associated with a longer retention time. *Y. enterocolitica* gut colonisation of apparently healthy animals (particularly pigs) at slaughter is well-recognised [8], and more prolonged excretion of Y. enterocolitica as compared with Y. pseudotuberculosis following infection in animals has been noted in vivo [73].

Competition for essential nutrients is increasingly recognised to be a survival strategy for pathogens [74]. Further metabolic evidence for Y. enterocolitica and Y. pseudotuberculosis occupying different niches while both being enteric pathogens is provided by the Y. enterocolitica–specific hydrogenase clusters. The two Y. enterocolitica [NiFe] hydrogenase operons are absent from the sequenced *Y. pestis* and Y. pseudotuberculosis genomes and appear as clear insertions into Y. enterocolitica. Furthermore, the notably compact arrangement of these clusters and the microarray data showing that all biotypes of Y. enterocolitica possess these genes suggest that they were acquired by lateral transfer at a point soon after speciation. H_2_ is abundant in the intestines and deeper tissues of animals and humans, a product of fermentative growth by colonic bacteria [54,55,75]. Since it has been shown that the ability to use H_2_ as an energy source for some enteric bacteria is central to their ability to colonise the gut and ultimately to cause disease [53,54], it is intriguing to consider why these functions are apparently unimportant for *Y. pseudotuberculosis,* also primarily a faecal–oral pathogen, and what that may suggest about differing disease processes in Y. enterocolitica and Y. pseudotuberculosis.

Wider comparisons with other members of the enterobacteriaceae have highlighted interesting parallels in their evolution. Like *Y. pestis, S. typhi* has become an acute systemic pathogen whilst its relatives, such as *S. typhimurium,* have remained essentially as enteropathogens. It is apparent that like *Yersinia, Salmonella* diversity is being driven by phage integration, plasmid acquisition (both integrated and extrachromosomal), and pseudogene formation (and gene deletion), as well as through the introduction of novel DNA through flexible loci, such as tRNA genes. In addition to these general themes, there are some more specific overlaps. It has been previously shown that the S. typhi plasmid, pHCM2, is highly related to the Y. pestis plasmid, pMT1 [19], and it is thought that the pathogenicity island, SPI-7 (encoding the major virulence antigen genes), and the important *Yersinia* YAPI loci [41,76] were derived from a common ancestor [43]. This suggests DNA exchange or that *Salmonella* and *Yersinia* have shared a common gene pool.

There is also similar evidence of metabolic “streamlining” in *Salmonella*. We have highlighted several functions that have been lost by one or more of the yersiniae, including the Y. enterocolitica–specific *ttr* cluster and *cob*/*pdu* operons, conferring the ability to use completely different energy supplies in and around the gut [31], as well as the cellulose biosynthetic cluster (discussed above). Similar observations can be made when comparing S. typhi with S. typhimurium. For example, the *cob*/*pdu*, *ttr,* and the *cel* gene clusters all carry multiple pseudogenes in *S. typhi,* yet have all been maintained apparently intact by S. typhimurium. Other similarities relate to hydrogenase gene clusters. In *S. typhi,* there is evidence of gene loss within hydrogenase clusters, with pseudogenes present in the *hya* (hydrogenase 1) and the membrane-bound hydrogenase gene cluster.

These data imply that members of the yersiniae and salmonellae have found common solutions to niche adaptation by gene acquisition and loss, perhaps even occupying similar metabolic niches. Moreover, as in S. typhi and *S. typhimurium,* although Y. enterocolitica and Y. pseudotuberculosis are both enteric pathogens, localisation and dynamics of Y. enterocolitica infection, we predict, in terms of site and rate of maximal growth in the host, are significantly different from Y. pseudotuberculosis.

## Materials and Methods

We chose to sequence a human septicaemia isolate, Y. enterocolitica strain 8081 [77]. 8081 is the prototype Y. enterocolitica strain that has been used extensively in the murine yersiniosis infection model to study gastrointestinal host–pathogen interactions and has been developed as an effective oral vaccine delivery system [78–80]. A single colony of Y. enterocolitica strain 8081 was picked from Congo Red agar and grown overnight in BAB broth with shaking at 30 °C. Cells were collected and total DNA (10 mg) was isolated using proteinase K treatment followed by phenol extraction. The DNA was fragmented by sonication, and several libraries were generated in pUC18 using size fractions ranging from 1.0 to 2.5 kb. The whole genome was sequenced to a depth of 9× coverage from M13mp18 (insert size 1.4–2 kb) and pUC18 (insert size 2.2–4.2 kb) small-insert libraries using dye-terminator chemistry on ABI3700 automated sequencers. End sequences from larger insert plasmid (pBACe3.6, 12–30 kb insert size) libraries were used as a scaffold.

The sequence was assembled, finished, and annotated as described previously [81], using the program Artemis [82] to collate data and facilitate annotation.

The genome sequences of *Y. enterocolitica, Y. pestis* strain CO92, Y. pestis strain KIM10+, Y. pestis strain 91001, and Y. pseudotuberculosis strain IP32953 were compared pairwise using the Artemis Comparison Tool [83]. Pseudogenes had one or more mutations that would ablate expression; each of the inactivating mutations was subsequently checked against the original sequencing data.

The pYVe8081 virulence plasmid (67,721 bp) was also sequenced as part of the genomic shotgun. The sequence of this plasmid was found to be identical to that previously sequenced [84], apart from a single-base insertion and nine single-nucleotide differences, seven of which were synonymous or located in noncoding regions. The nonsynonymous mutations were found in YEP0063 (hypothetical protein, Phe–Leu substitution) and YEP0069 (transposase, Phe–Ser substitution) (unpublished data).

The genome has been submitted to the EMBL public database (10.1371/journal.pgen.0020206_01). Accession numbers are listed in the Supporting Information section below. The genome submission is MIGS compliant (10.1371/journal.pgen.0020206_02, GCAT identifier 000001_GCAT). This strain has been deposited as NCTC 13174.

### Generating orthologous gene sets.

Orthologous gene sets were identified by reciprocal FASTA searches. Only those pairs of homologous CDSs were retained for further analysis where the predicted amino acid identity was ≥40% over 80% of the protein length. These genes were then subjected to manual curation using gene synteny to increase the accuracy of this analysis. This strategy was applied to pairwise comparisons of the genomes of Y. enterocolitica strain 8081, Y. pestis (strains CO92, 91001, and KIM10+), and Y. pseudotuberculosis strain IP32953.

### Microarray analysis.

The microarray was designed to include all 4,036 predicted CDSs from the Y. enterocolitica 8081 genome as previously described [69]. The strains and raw microarray data used in this study was derived from a much larger phylogenomic study using a microarray based on Y. enterocolitica strain 8081 [69]. Data was processed and genes designated as present, divergent, or absent (highly divergent) as previously described [69]. Table S2 details the strains used in this study. All strains were identified using standard biochemical typing tests as previously described [69]. Microarray data can be found in Array Express (http://www.ebi.ac.uk/arrayexpress) in Supporting Information below.

## Supporting Information

Table S1 Insertion Sequence Elements Found within the Sequenced *Yersinia* Genomes (100 KB DOC)

Table S2 Strain List of the Y. enterocolitica Isolates Used in the Microarray Analysis (92 KB DOC)

Table S3 CDS Unique to Y. enterocolitica Strain 8081 as Defined by Comparative Microarray Analysis of 33 Other Isolates of Biotypes 1B, 1A, 2, 3, and 4 (224 KB DOC)

### Accession Numbers

The EMBL (10.1371/journal.pgen.0020206_01) accession numbers for the genome of Y. enterocolitica strain 8081are AM286415 and AM286416 (plasmid).

The Array Express (10.1371/journal.pgen.0020206_03) accession number for the Y. enterocolitica strain 8081 microarray data is E-BUGS-36. Y. enterocolitica strain 8081 was deposited with the Health Protection Agency (United Kingdom, http://www.hpa.org.uk/nctc/searcher.html) as NCTC 13174.

## Abbreviations

bp: base pair
CDS: coding sequence
CRISPR: clustered regularly interspaced short palindromic repeats
GSP: general secretion pathway
kb: kilobase
MTA: methylthioadenosine
OPG: osmoregulated periplasmic glucan
pYV: *Yersinia* virulence plasmid
PZ: plasticity zone
TTSS: type three secretion system
YGI-1: **Yersinia** genomic island 1
YGI-2: *Yersinia* genomic island 2
YGI-3: *Yersinia* genomic island 3
YGI-4: *Yersinia* genomic island 4
