# Supplementary material for: The Complete Genome Sequence and Comparative Genome Analysis of the High Pathogenicity Yersinia enterocolitica Strain 8081
Source: PLoS Genet. 2006 Dec 15;2(12):e206. doi: 10.1371/journal.pgen.0020206 (PMC1698947; doi:10.1371/journal.pgen.0020206)
Supplement: Supplementary file 1 [file pgen.0020206.st001.doc]

Table S1. Insertion sequence elements found within the sequenced *Yersinia* genomes.

|  | *Y. en* | *Y. pestis* | | | *Y. pstb* | Comments^5^ |
| --- | --- | --- | --- | --- | --- | --- |
|  | **Strain** | | | | |  |
| **IS name** | 8081 | CO92^1^ | KIM10+^2^ | 91001^3^ | IP32953^4^ |  |
| IS3 | 2 | 0 | 0 | 0 | 0 |  |
| IS100 | 0 | 44 | 35 | 30 | 5 |  |
| IS1222 | 1 | 1 | 0 | 0 | 0 |  |
| IS285 | 1 | 21 | 19 | 23 | 7 |  |
| IS1328 | 3, 2* (2*) | 0 | 0 | 0 | 0 |  |
| IS1329 | 4, 2* | 0 | 0 | 0 | 0 |  |
| IS1330 | 7, 1* | 0 | 0 | 0 | 0 |  |
| IS1400 | 2, 2* (2*) | 0 | 0 | 0 | 0 |  |
| IS1541 | 3 (1) | 62, 2* | 49, 9* | 43, 5* | 5 |  |
| IS1660 | 6 (1) | 0 | 0 | 0 | 0 | Novel, 89% to closest: IS4 |
| IS1661 | 0 | 9 | 8, 2* | 7, 1* | 3 | Novel, 39% to closest: IS1397 |
| IS1664 | 3 | 0 | 0 | 0 | 0 | Novel, 40% to closest: IS1661 |
| IS1665 | 5 (1) | 0 | 0 | 0 | 0 | Novel, IS4-like. Highly similar to IS1660 same IR and almost identical apart from a possible programmed frameshift. |
| IS1666 | 1 | 0 | 0 | 0 | 0 | Novel, 39 % to closest: IS1329 |
| IS1667 | 7, 1* | 0 | 0 | 0 | 0 | Novel, 44% to closest: IS1328 |
| IS1668 | 2 | 0 | 0 | 0 | 0 | Novel, 46% Thermobacteria IS |
| IS1669 | 5 (2) | 0 | 0 | 0 | 0 | Novel, 66% to closest *Pseudomonas* IS element |
| Total | 60 (9) | 139 | 122 | 109 | 20 |  |

^1^ Parkhill *et al*., [19]; ^2^ Deng *et al*., [21]; ^3^ Song *et al*., [20]; ^4^ Chain *et al*., [18]; ^5^ % refer to percentage nucleotide identity.

* partial sequences

Numbers in brackets indicate number of IS elements on the strain 8081 plasmid pYVe8081.

*Y. en – Y. enterocolitica*

*Y. pstb – Y. pseudotuberculosis*

IS - insertion sequence element.

IR - inverted repeat sequence.
