# Supplementary material for: The Complete Genome Sequence and Comparative Genome Analysis of the High Pathogenicity Yersinia enterocolitica Strain 8081
Source: PLoS Genet. 2006 Dec 15;2(12):e206. doi: 10.1371/journal.pgen.0020206 (PMC1698947; doi:10.1371/journal.pgen.0020206)
Supplement: Supplementary file 2 [file pgen.0020206.st002.doc]

Table S2. Strain list of the *Y. enterocolitica* isolates used in the microarray analysis.

| **Strain name** | **host** | **serotype** | **biotype** | **isolated** |
| --- | --- | --- | --- | --- |
| 8081 | HUMAN | O8 | 1B | Human clinical isolate |
| Y30 | HUMAN | O8 | 1B | Human walter Hill |
| Y68 | HUMAN | O20 | 1B | J Farmer CDC |
| Y69 | HUMAN | O21 | 1B | J Farmer CDC |
| Y70 | HUMAN | O18 | 1B | Human leg wound J Farmer CDC |
| Y71 | HUMAN | O4,32 | 1B | J Farmer CDC |
| Y73 | HUMAN | O20 | 1B | Human fecal J Farmer CDC |
| Y89 | HUMAN | O8 | 1B | Human thumb J Farmer CDC |
| 212/02 | PIG | O9 | 2 | VLA abattior |
| 119/02 | SHEEP | O9 | 2 | VLA abattior |
| 153/02 | CATTLE | O5,27 | 3 | VLA abattior |
| 177/02 | CATTLE | O5,27 | 3 | VLA abattior |
| 16/03 | HUMAN | O5 | 3 | HPA blood |
| 56/03 | HUMAN | O9 | 3 | IID GP CASE |
| 202/02 | PIG | O5 | 3 | VLA abattior |
| 218/02 | PIG | O9 | 3 | VLA abattior |
| 231/02 | PIG | O5,27 | 3 | VLA abattior |
| 149/02 | SHEEP | O5,27 | 3 | VLA abattior |
| 7/03 | HUMAN | O3 | 4 | HPA faeces |
| 8/03 | HUMAN | O3 | 4 | HPA faeces |
| 190/02 | HUMAN | O3 | 4 | HPA Not recorded |
| 201/02 | PIG | O3 | 4 | VLA abattior |
| 209/02 | PIG | O5,27 | 4 | VLA abattior |
| 220/02 | PIG | O3 | 4 | VLA abattior |
| 227/02 | PIG | O3 | 4 | VLA abattior |
| 135/02 | SHEEP | O3 | 4 | VLA abattior |
| 14/02 | CATTLE | O6,30 | 1A | VLA abattior |
| 81/02 | CATTLE | O6,30 | 1A | VLA abattior |
| 9/03 | HUMAN | O6,30 | 1A | HPA faeces |
| 35/03 | HUMAN | 0? | 1A | IID GP CASE |
| 208/02 | PIG | O5 | 1A | VLA abattior |
| 77/03 | PIG | O19,8 | 1A | Not recorded |
| 12/02 | SHEEP | O19,8 | 1A | VLA abattior |
| 30/02 | SHEEP | O6,30 | 1A | VLA abattior |
