# Supplementary material for: The Complete Genome Sequence and Comparative Genome Analysis of the High Pathogenicity Yersinia enterocolitica Strain 8081
Source: PLoS Genet. 2006 Dec 15;2(12):e206. doi: 10.1371/journal.pgen.0020206 (PMC1698947; doi:10.1371/journal.pgen.0020206)
Supplement: Supplementary file 3 [file pgen.0020206.st003.doc]

Table S3. CDS unique to *Y. enterocolitica* strain 8081 as defined by comparative microarray analysis of 33 other isolates of biotypes 1B, 1A, 2, 3 and 4.

| **Systematic**  **ID** | **Genename** | **Frame** | **Start** | **End** | **Size (aa)** | **Product description** |
| --- | --- | --- | --- | --- | --- | --- |

1. YGI-3

| YE0974 | *-* | - | 1096884 | 1097051 | 56 | prophage integrase (partial) |
| --- | --- | --- | --- | --- | --- | --- |
| YE0975 | *-* | + | 1097520 | 1098374 | 284 | conserved hypothetical protein |
| YE0976 | *-* | + | 1098715 | 1099029 | 104 | hypothetical protein |
| YE0977 | *-* | + | 1099313 | 1100518 | 401 | putative integrase/recombinase |
| YE0978 | *-* | + | 1100515 | 1101873 | 452 | putative integrase/recombinase |
| YE0979 | *-* | + | 1101870 | 1103729 | 619 | conserved DNA-binding protein |
| YE0980 | *-* | + | 1103765 | 1104169 | 134 | conserved hypothetical protein |
| YE0981 | *-* | + | 1104276 | 1104698 | 140 | hypothetical protein |
| YE0982 | *-* | - | 1104747 | 1105136 | 129 | putative lipoprotein |
| YE0983 | *-* | - | 1105166 | 1105807 | 213 | putative membrane protein |
| YE0984 | *-* | - | 1106028 | 1107299 | 423 | repetative plasmid-related protein |
| YE0986 | *-* | - | 1107316 | 1107732 | 138 | putative ATP/GTP-binding protein |
| YE0987 | *-* | - | 1108214 | 1108765 | 183 | putative inner membrane protein |
| YE0988 | *-* | - | 1108807 | 1110333 | 508 | conserved hypothetical protein |
| YE0989 | *-* | + | 1110505 | 1110981 | 158 | hypothetical protein |
| YE0989 | *-* | + | 1110505 | 1110981 | 158 | hypothetical protein |
| YE0990 | *-* | - | 1111058 | 1112893 | 611 | AYP/GTP-binding protein |
| YE0991 | *-* | - | 1112875 | 1113297 | 140 | hypothetical protein |
| YE0992 | *-* | - | 1113318 | 1114508 | 396 | hypothetical protein |
| YE0993 | *-* | - | 1114774 | 1116003 | 409 | putative prophage integrase |

**2. Prophage YE250**

| YE2292 | *-* | - | 2504021 | 2504479 | 152 | putative phage tail fiber assembly protein |
| --- | --- | --- | --- | --- | --- | --- |
| YE2293 | *-* | - | 2504488 | 2506299 | 603 | putative phage tail protein |
| YE2296 | *-* | - | 2507298 | 2510480 | 1060 | putative phage host specificity protein |
| YE2297 | *-* | + | 2510655 | 2510954 | 99 | hypothetical phage protein |
| YE2299 | *-* | - | 2511244 | 2511846 | 200 | putative phage tail assembly protein |
| YE2300 | *-* | - | 2511908 | 2512330 | 140 | hypothetical phage protein |
| YE2301 | *-* | - | 2512327 | 2512725 | 132 | phage-related membrane protein |
| YE2302 | *-* | - | 2512818 | 2513546 | 242 | hypothetical phage protein |
| YE2303 | *-* | - | 2513558 | 2513896 | 112 | hypothetical phage protein |
| YE2305 | *-* | + | 2514312 | 2514725 | 137 | hypothetical phage protein |
| YE2306 | *-* | - | 2514836 | 2515153 | 105 | hypothetical phage protein |
| YE2310 | *-* | - | 2515867 | 2516571 | 234 | putative phage minor tail protein |
| YE2311 | *-* | - | 2516574 | 2517326 | 250 | putative phage protein |
| YE2312 | *-* | - | 2517343 | 2517684 | 113 | putative phage protein |
| YE2313 | *-* | - | 2517687 | 2520482 | 931 | putative phage tail protein |
| YE2314 | *-* | - | 2520735 | 2520914 | 59 | hypothetical phage protein |
| YE2315 | *-* | - | 2521043 | 2521354 | 103 | hypothetical phage protein |
| YE2316 | *-* | - | 2521367 | 2522287 | 306 | putative phage protein |
| YE2317 | *-* | - | 2522354 | 2522761 | 135 | hypothetical phage protein |
| YE2318 | *-* | - | 2522758 | 2523342 | 194 | hypothetical phage protein |
| YE2319 | *-* | - | 2523344 | 2523694 | 116 | hypothetical phage protein |
| YE2320 | *-* | - | 2523694 | 2524221 | 175 | hypothetical phage protein |
| YE2321 | *-* | - | 2524224 | 2525429 | 401 | putative phage protein |
| YE2322 | *-* | - | 2525443 | 2526216 | 257 | hypothetical phage protein |
| YE2323 | *-* | - | 2526338 | 2527450 | 370 | hypothetical phage protein |
| YE2324 | *-* | - | 2527451 | 2528839 | 462 | hypothetical phage protein |
| YE2325 | *-* | - | 2528839 | 2530401 | 520 | putative phage terminase large subunit |
| YE2326 | *-* | - | 2530398 | 2530970 | 190 | putative phage terminase, small subunit |
| YE2327 | *-* | + | 2530999 | 2531142 | 47 | hypothetical phage protein |
| YE2328 | *-* | - | 2531348 | 2531527 | 59 | hypothetical phage protein |
| YE2329 | *-* | + | 2531679 | 2532017 | 112 | hypothetical phage protein |
| YE2331 | *-* | - | 2532181 | 2532870 | 229 | hypothetical phage-related protein |
| YE2335 | *-* | - | 2534688 | 2535218 | 176 | hypothetical phage protein |
| YE2336 | *-* | - | 2535454 | 2535942 | 162 | putative late gene antiterminator protein |
| YE2339 | *-* | - | 2536905 | 2537351 | 148 | NinB-like phage protein |
| YE2340 | *-* | - | 2537344 | 2537745 | 133 | hypothetical phage protein |
| YE2341 | *-* | - | 2537742 | 2538044 | 100 | hypothetical phage protein |
| YE2343 | *-* | + | 2538309 | 2538902 | 197 | hypothetical phage protein |
| YE2344 | *-* | - | 2539004 | 2539240 | 78 | hypothetical phage protein |
| YE2345 | *-* | - | 2539237 | 2539932 | 231 | DNA replication protein |
| YE2346 | *-* | - | 2539929 | 2540828 | 299 | putative DNA-binding phage protein |
| YE2347 | *-* | - | 2540825 | 2541490 | 221 | hypothetical phage related protein |
| YE2350 | *cI* | + | 2542568 | 2543167 | 199 | repressor protein CI |
| YE2351 | *-* | - | 2544069 | 2544380 | 103 | IS-element protein (fragment) |
| YE2352 | *-* | + | 2544663 | 2545238 | 191 | hypothetical phage protein |
| YE2353 | *-* | + | 2545366 | 2545776 | 136 | hypothetical phage protein |
| YE2355 | *-* | + | 2546079 | 2546744 | 221 | exodeoxyribonuclease |
| YE2357 | *-* | + | 2547409 | 2548086 | 225 | hypothetical phage protein |
| YE2358 | *-* | + | 2548482 | 2548850 | 122 | hypothetical exported phage protein |
| YE2359 | *-* | + | 2549000 | 2549503 | 167 | hypothetical phage protein |
| YE2361 | *dam* | + | 2550161 | 2551021 | 286 | putative adenine-specific modification methylase |
| YE2362 | *dcm* | + | 2551018 | 2552763 | 581 | putative cytosine-specific modification methylase |
| YE2363 | *-* | + | 2553100 | 2553348 | 82 | putative phage excisionase |

**3. PZ**

| YE3454 | *-* | - | 3765558 | 3767960 | 800 | putative hemolysin |
| --- | --- | --- | --- | --- | --- | --- |
| YE3462 | *-* | + | 3775095 | 3776192 | 365 | hypothetical protein |
| YE3463 | *-* | + | 3776189 | 3777172 | 327 | hypothetical protein |
| YE3464 | *-* | + | 3777495 | 3777758 | 87 | hypothetical protein |
| YE3465 | *-* | - | 3777818 | 3779338 | 506 | putative membrane protein |
| YE3466 | *-* | - | 3779338 | 3780744 | 468 | putative exported protein |
| YE3467 | *-* | - | 3780765 | 3781718 | 317 | putative exported protein |
| YE3468 | *-* | - | 3781715 | 3782068 | 117 | hypothetical protein |
| YE3472 | *arsR2* | + | 3783946 | 3784299 | 117 | arsenical resistance operon repressor |
| YE3473 | *arsA2* | + | 3784726 | 3786477 | 583 | arsenical pump-driving ATPase |
| YE3474 | *arsB2* | + | 3786526 | 3787815 | 429 | arsenical pump membrane protein |
| YE3475 | *arsC2* | + | 3787828 | 3788253 | 141 | arsenate reductase |
| YE3477 | *arsH* | - | 3789694 | 3790404 | 236 | putative arsenic resistance protein |
| YE3478 | *-* | - | 3790536 | 3790829 | 97 | conserved hypothetical protein |
| YE3479 | *-* | + | 3790936 | 3792069 | 377 | putative membrane transport protein |
| YE3480 | *ccdB* | - | 3792208 | 3792522 | 104 | cytotoxic protein |
| YE3481 | *ccdA* | - | 3792525 | 3792761 | 78 | gyrase inhibitor antitoxin |
| YE3482 | *-* | - | 3793238 | 3795880 | 880 | Conserved hypothetical protein |
| YE3483 | *-* | - | 3796042 | 3796446 | 134 | putative lipoprotein |
| YE3484 | *-* | - | 3796456 | 3797913 | 485 | conserved hypothetical protein |
| YE3485 | *-* | - | 3797961 | 3798827 | 288 | putative exported protein |
| YE3486 | *-* | - | 3798824 | 3799477 | 217 | putative membrane protein |
| YE3487 | *-* | - | 3799474 | 3799848 | 124 | putative inner membrane protein |
| YE3488 | *-* | - | 3799858 | 3800208 | 116 | putative exported protein |
| YE3489 | *-* | - | 3800224 | 3800472 | 82 | putative membrane protein |
| YE3490 | *-* | - | 3800469 | 3800777 | 102 | putative exported protein |
| YE3492 | *-* | - | 3801682 | 3802434 | 250 | putative inner membrane protein |
| YE3493 | *-* | - | 3802434 | 3804554 | 706 | TraG-family protein |
| YE3495 | *-* | - | 3805875 | 3806642 | 255 | putative membrane protein |
| YE3496 | *-* | - | 3807111 | 3807365 | 84 | hypothetical protein |
| YE3497 | *-* | - | 3808031 | 3808951 | 306 | putative transposase |

**4. Non-clustered CDS**

| YE0124 | *-* | + | 141797 | 142450 | 217 | probable hemophore |
| --- | --- | --- | --- | --- | --- | --- |
| YE0147 | *-* | - | 172798 | 174321 | 507 | putative magnesium chelatase family protein |
| YE1644 | *gpD* | - | 1849792 | 1850901 | 369 | regulator of late gene expression |
| YE3124 | *-* | + | 3397227 | 3398267 | 346 | putative Pyridoxal-phosphate dependent protein |
| YE3251 | *-* | + | 3546281 | 3546622 | 113 | putative exported protein |
| YE3287 | *dapD* | + | 3590890 | 3591714 | 274 | 2,3,4,5-tetrahydropyridine-2-carboxylate N-succinyltransferase |
| YE3509 | *-* | - | 3820501 | 3821043 | 180 | hypothetical protein |
| YE3511 | *-* | - | 3822260 | 3823462 | 400 | conserved hypothetical protein |
| YE3513 | *-* | - | 3824134 | 3825720 | 528 | putative ATP/GTP binding protein |
| YE3514 | *dnaB2* | - | 3825720 | 3827087 | 455 | putative DNA helicase |
| YE4064 | *-* | + | 4433428 | 4433628 | 66 | putative tautomerase |
| YE4070 | *-* | - | 4440935 | 4441639 | 234 | putative exported protein |
| YE4171 | *recF* | - | 4570452 | 4571537 | 361 | DNA metabolism protein |
| YE4178 | *thdF* | + | 4577454 | 4578818 | 454 | putative thiophene and furan oxidation protein |
| YE4199 | *pstA* | - | 4597494 | 4598381 | 295 | putative phosphate transport system permease |
